# A DNA Vaccine against Chikungunya Virus Is Protective in Mice and Induces Neutralizing Antibodies in Mice and Nonhuman Primates

**DOI:** 10.1371/journal.pntd.0000928

**Published:** 2011-01-11

**Authors:** Karthik Mallilankaraman, Devon J. Shedlock, Huihui Bao, Omkar U. Kawalekar, Paolo Fagone, Aarthi A. Ramanathan, Bernadette Ferraro, Jennifer Stabenow, Paluru Vijayachari, Senthil G. Sundaram, Nagarajan Muruganandam, Gopalsamy Sarangan, Padma Srikanth, Amir S. Khan, Mark G. Lewis, J. Joseph Kim, Niranjan Y. Sardesai, Karuppiah Muthumani, David B. Weiner

**Affiliations:** 1 Department of Pathology and Laboratory Medicine, University of Pennsylvania School of Medicine, Philadelphia, Pennsylvania, United States of America; 2 Regional Biocontainment Lab, University of Tennessee Health Science Center, Memphis, Tennessee, United States of America; 3 Regional Medical Research Centers, Indian Council of Medical Research, Port Blair, Andaman & Nicobar Islands, India; 4 Department of Microbiology, Sri Ramachandra Medical College & Research Institute, Chennai, India; 5 Inovio Pharmaceuticals, Blue Bell, Pennsylvania, United States of America; 6 Bioqual Inc, Rockville, Maryland, United States of America; Centers for Disease Control and Prevention, United States of America

## Abstract

Chikungunya virus (CHIKV) is an emerging mosquito-borne alphavirus indigenous to tropical Africa and Asia. Acute illness is characterized by fever, arthralgias, conjunctivitis, rash, and sometimes arthritis. Relatively little is known about the antigenic targets for immunity, and no licensed vaccines or therapeutics are currently available for the pathogen. While the *Aedes aegypti* mosquito is its primary vector, recent evidence suggests that other carriers can transmit CHIKV thus raising concerns about its spread outside of natural endemic areas to new countries including the U.S. and Europe. Considering the potential for pandemic spread, understanding the development of immunity is paramount to the development of effective counter measures against CHIKV. In this study, we isolated a new CHIKV virus from an acutely infected human patient and developed a defined viral challenge stock in mice that allowed us to study viral pathogenesis and develop a viral neutralization assay. We then constructed a synthetic DNA vaccine delivered by *in vivo* electroporation (EP) that expresses a component of the CHIKV envelope glycoprotein and used this model to evaluate its efficacy. Vaccination induced robust antigen-specific cellular and humoral immune responses, which individually were capable of providing protection against CHIKV challenge in mice. Furthermore, vaccine studies in rhesus macaques demonstrated induction of nAb responses, which mimicked those induced in convalescent human patient sera. These data suggest a protective role for nAb against CHIKV disease and support further study of envelope-based CHIKV DNA vaccines.

## Author Summary

Chikungunya fever epidemics are sustained by a cycle of human-mosquito-human transmission, with the epidemic cycle being similar to those of dengue and urban yellow fever. While the threat of a pandemic continues to engage the public's attention, the peculiar problems associated with the more immediate and very real seasonal epidemics are also worthy of consideration. Specifically, there are limited viral strains that have been characterized and available for laboratory study as well as limited knowledge of immune responses induced to the virus. In this study, we isolated CHIKV virus from an acutely infected human patient and used this new virus to develop a neutralization assay and a challenge stock, which is effective in a mouse model. Furthermore, we analyzed the ability of an envelope-based synthetic DNA-based vaccine to impact viral disease in the mouse model and to generate protective levels of immune responses in nonhuman primates. We observed that this novel vaccine approach generated protective levels of immune responses in both mouse and non-human primate models. We believe that these studies advance the field of Chikungunya vaccine research as well as the study of immune protection to CHIKV.

## Introduction

Chikungunya virus (CHIKV) is an enveloped, single-stranded, positive-sense RNA virus that belongs to the family Togaviridae, genus Alphavirus, and is part of the Semliki Forest virus antigenic complex [1], [2], [3], [4]. CHIKV has been responsible for unprecedented, explosive outbreaks during 2004 and 2007 in India and the Indian Ocean islands [2], [4], [5], [6], [7]. These outbreaks represent the largest documented cases associated with the virus [8]. Chikungunya fever, the disease caused by CHIKV, was first recognized in epidemic form in East Africa in 1952-1953 [3], [9] and the viral agent was first isolated at that time from the blood of a febrile patient in Tanzania [9]. In the local Swahili dialect, “Chikungunya” means “stooping” or “bending”, which describes the physical position often assumed by CHIKV-infected patients [3], [9], [10]. Since that time, CHIKV has been identified as the agent responsible for major epidemics in both Africa and Southeast Asia and continues to be a re-emerging agent of great interest to public health [1], [11], [12], [13]. Despite its importance as an emerging virus and potential biological weapon, there are no specific licensed vaccines or antiviral treatments for Chikungunya.

Currently, CHIKV is geographically distributed from Africa through Southeast Asia and South America and is principally transmitted to humans through *Aedes* mosquitoes [2], [14]. Recently, a mutation in the CHIKV envelope (E1-A226V) was found to be directly responsible for the significant recent increase in CHIKV infectivity, and studies confirmed that this single amino acid substitution can influence vector specificity. This finding provides a plausible explanation of how this variant virus caused an epidemic in a region lacking the normal insect vector *Ae. Aegypti* [15], [16].

While Chikungunya is not typically associated with human mortality, epidemics often present public health threats due to substantial morbidity, suffering, and loss of economic productivity. The incubation period of the virus ranges between 1–2 weeks and infected individuals usually experience an acute illness with fever, headache, rash, nausea, vomiting, incapacitating polyarthralgia, severe muscle pain, and joint stiffness [17]. The most prominent clinical feature of CHIKV disease is arthralgia, which can be debilitating and prolonged [5], [17], [18], [19], [20]. Though the pathogenesis of the virus in humans is not exactly clear, recent findings of CHIKV infection in muscle tissue and macrophages could explain some features of its clinical manifestations [12], [18], [21], [22], [23]. Due to these characteristic clinical symptoms of infection, outbreaks of CHIKV have devastating public health and economic effects.

The first reported outbreak of Chikungunya occurred on Lamu Island, Kenya, in 2004. Later the virus spread to La Reunion Island, infecting more than two hundred thousand individuals [11], then to other islands in the Indian Ocean [24], and then finally into India in 2006 [25]. Furthermore, the La Reunion isolate outbreaks were associated with unexpected morbidity [26]. Importantly, exposed travelers returning from the affected areas to Europe, the US, Canada, Hong Kong, and numerous other countries have carried the virus into these new niches where the imported cases were subsequently identified [8]. Although these instances of viral importation were effectively controlled, they serve as a reminder of how easily this agent could be introduced and spread into new geographical locations including industrialized nations.

Thus far, while several vaccines have been developed against this disease, such as a formalin inactivated vaccine [27], a virus-like particle vaccine [28] and a live attenuated vaccine [29], none have advanced to clinical development and therefore illustrate an important area of need. Recently, a novel consensus-based DNA vaccine was developed by our laboratory and reported to be immunogenic in mice [30], inducing both measurable cellular and humoral immune responses. However, the neutralizing and hemagglutination-inhibiting antibody responses were not examined. While nAb to CHIKV during natural infection in humans are not well understood, recent sero-surveys during outbreaks suggest a protective role for prevention of replication [23], [28], [30], [31], [32]. Furthermore, prior infection is thought to be protective against subsequent CHIKV infection. Therefore, by examination of nAb titers during natural infection in humans, a benchmark for vaccine development in this study, we aimed to establish a correlation with these responses and protection.

Accordingly, we isolated a new viral isolate from an acutely infected patient, and termed PENN CHIKV-2008 (PC-08), and characterized its biology in mice, and also used it to develop an *in vitro* neutralization assay. Furthermore, we modified our previous DNA vaccine to optimize for the capacity of neutralization by designing a single consensus envelope DNA vaccine construct expressing all three envelope proteins. We compared its effects in mice with a CTL-only-inducing Capsid vaccine in a challenge model. Finally, we compared in non-human primates vaccine-induced immune responses with human CHIKV convalescent sera as a measure of protective immunity.

## Materials and Methods

### Ethics statement

Samples used in the study were provided by Regional Medical Research Center (RMRC), Indian Council of Medical Research (ICMR), Port Blair, India and Sri Ramachandra Medical College & Research Institute (SRMC&RI), Chennai, India. These samples were previously obtained with proper informed consent at the respective institutions. The collected samples were coded and stored. The samples do not contain identifying information regarding the patients that donated the samples and under an agreement between the collaborating institutions that determined at no point was the key decoding patient data disclosed to the investigators performing the assays. The study was reviewed by the respective Institutional Human Ethical Committees and approvals were obtained. The de-identified samples were transported to University of Pennsylvania, PA, USA following EHRS guidelines and after obtaining the CDC import permit to Import or Transport Etiologic Agents, Hosts or Vectors of Human Disease (Permit # 2008-03-027). Appropriate practices and procedures as defined in the Biosafety in microbiological and biomedical laboratories (US Dept. of Health and Human Services) were used in sample handling. Samples were stored at −80°C in a bio safety level-3 (BSL-3) facility at the University of Pennsylvania, PA, USA.

Primate studies were conducted by the subcontract at Bioqual Inc, MD. The animal management program of this institution is accredited by the American Association for the Accreditation of laboratory Animal Care and meets NIH standards as outlined the in the Guide and care and use of laboratory animals. This institution also accepts as mandatory PHS policy on Humane Care of Vertebrate Animals used in testing, research and training.

### Cell culture and animals

Vero 76 (ATCC CRL-1587) and RD (ATCC CCL-136) cells were cultured in complete medium (Eagle's Minimum Essential Medium) containing 10% fetal bovine serum, 1 mM glutamine, 1 mM sodium pyruvate, 100 U/ml penicillin and 100 µg/ml streptomycin. Cells were incubated in a 5% CO_2_ humidified incubator at 37°C [33]. 8-week-old female BALB/c mice (Jackson laboratories, Indianapolis, IN) were used in these experiments. All animals were housed in a temperature-controlled, light-cycled facility in accordance with the guidelines of the National Institutes of Health (Bethesda, MD, USA) and the University of Pennsylvania Institutional Animal Care and Use Committee (IACUC). Rhesus macaques (*Macaca mulatta*), aged 4–8 years, were housed at Bioqual, Inc, Rockville, MD 20850. The experiments reported herein were conducted according to the principles set forth in the Guide for the Care and Use of Laboratory Animals, Institute of Laboratory Animal Resources, National Research Council, Department of Health and Human Services (DHHS) Publication number NIH-86-23 (1985).

### DNA vaccine construction and expression

The CHIKV DNA constructs pCHIKV-E1, pCHIKV-E2, and pCHIKV-Capsid have been previously described [30]. The combined CHIKV-envelope construct was designed with the structural genes E3, E2 and E1 linked together in a single construct with furin cleavage sites between the individual genes [34]. The consensus gene sequences were constructed using the predicted consensus sequences from the sequences available in the NCBI Genbank database and designated as pMCE321. We note that while the 6K protein is also a structural constituent of envelope, we did not include it in our vaccine construct because we sought to create a minimal vaccine construct capable of inducing broadly protective immune responses. The primary role of 6K is postulated to function in the selection of lipids that interact with the transmembrane domains of the glycoproteins [35]. Consensus sequences were optimized for Env expression, including codon and RNA optimization (GeneArt, Regensburg, Germany), a novel leader sequence was added [36] as were furin cleavage sites to facilitate Env processing as previously published [34] and inserted into the pVax1 expression vector (Invitrogen, CA) and designated as pMCE321. DNA preparations were made at Aldevraon (Forgo, ND), as previously described and formulated at 10 mg/ml in water.

Expression of pMCE321 was verified by immunoblotting and immunofluorescence. Vero and RD cells (1×10^6^cells) were transfected with pMCE321 constructs using the Fugene transfection method (Roche, Indianapolis, IN). Forty-eight hours post-transfection, proteins were isolated using cell lysis buffer, fractionated on SDS-PAGE (12%), and transferred to nitrocellulose using iBlot Dry Blotting System (Invitrogen, CA, USA). Immunodetection was performed using SNAP i.d. Protein Detection System (Millipore, MA, USA) with specific mouse antiserum and the expressed proteins were visualized with horseradish peroxidase-conjugated goat anti-mouse IgG using an ECL detection system (Amersham Pharmacia Biotech, Piscataway, NJ) [33].

### Immunofluorescence and histopathology

For immunofluorescence, Vero cells (2×10^5^ cells) were seeded in 2-chamber tissue culture treated glass slides (BD Falcon, MA, USA) and transfected with pVax1-E1, pVax1-E2, and pMCE321 vaccine constructs or control pVax1 vector. Forty eight hours post-transfection, cells were fixed with 2% paraformaldehyde, blocked with Glycine/BSA, and then incubated overnight at 4°C with mouse anti-Env IgG antibodies (1∶500 dilution). Excess antibodies were washed off and the secondary antibody AlexaFluor 488-anti mouse IgG (Invitrogen, Molecular Probes, USA) was added and incubated for 2 hours at 37°C. The cells were counter stained with DAPI for visualizing the nucleus and fixed with fluoromount G (Electron Microscopy Sciences, PA, USA). The confocal images were acquired with Zeiss LSM510 META NLO Laser Scanning Confocal Microscope with Two Photon Excitation at the Biomedical Imaging Core, University of Pennsylvania, PA, USA.

For histopathology studies, naïve and CHIKV DNA vaccinated mice, challenged with the CHIKV isolate, were bled and sacrificed on day 14 p.i. Tissue samples (brain, heart, lung, liver, and kidney) were collected and fixed in 10% buffered formalin solution for 24 h, and then stored in 70% ethanol prior to embedding, sectioning, and staining using hematoxylin and eosin (H&E) stain [23], [37].

### CHIKV patients

CHIKV-infected patient sera samples were obtained from ICMR, Port Blair, India and SRMC&RI, Chennai, India. The number of days from onset of illness to sampling ranged from 1 to 14 d. All cases had complained of fever with median duration of 3–5 d. Other common symptoms included Chills and Rigors (23%), myalgias (6%), gastrointestinal symptoms of diarrhea, abdominal pain (20%), vomiting (20%), severe joint pain (3%) and headache (13%) (Table 1).

**Table 1:** Clinical observation in Chikungunya patients.

Symptoms	# of Patients	%
Fever	30	100
Chills and Rigors	7	23
Head Ache	4	13
Myalgia	2	6
Rash/Hemorrhage	0	0
Severe Joint pain	1	3
Abdominal pain	6	20
Vomiting	6	20
RT-PCR positivity	13	43

### CHIK virus extraction

The isolation of virus from patient sera was carried out in Vero cells grown to 90–95% confluence in Eagle's complete medium (MEM) in T-25 tissue culture flasks (BD Falcon, USA). Patient serum (100 µl) was mixed with MEM (400 µl), adsorbed onto the cell culture (after removing the growth medium) for 2 hrs at 37°C, and then replenished with complete growth medium following washing with MEM. The inoculated cells were incubated at 37°C with 5% CO_2_ for 5 to 7 days and monitored daily for the development of cytopathic effects (CPE). When CPE was observed in more than 90% of the cells, the flasks were frozen at −80°C, freeze–thawed five times to facilitate cell lysis and virus release, and then centrifuged at 3,000rpm for 10 min to remove cellular debris. The isolate was then passaged five times in Vero cells, titrated, and stored at −80°C. The virus stock was designated as PC-08 (PENN CHIKV strain - 08).

Viral RNA from patient serum was extracted using QIAamp Viral RNA mini kit according to manufacturer's instructions (Qiagen Inc, Valencia, CA). A one-step RT-PCR test was carried out using Qiagen One step RT-PCR kit on a block thermo cycler (PTC-100, MJ Research, Waltham, MA, USA); 5 pmoles of each primer (CHIK-forward 5′-TATCCTGACCACCCAACGCTCC-3′ and CHIK-reverse 5′-ACATGCACATCC CACCTGCC-3′ amplify a 305 bp region within the gene coding for the viral envelope protein E2) were used with 10 µl 5X RT PCR buffer, 2 µl dNTP mix, 5 µl Q solution, 1 µl enzyme mix, 25 µl water, and 5 µl extracted RNA for a total reaction volume of 50 µl [38]. Thermocycler conditions were as follows: 50°C for 30 min, 94°C for 2 min, then 40 cycles of 94°C for 15 s, 55°C for 30 s and 68°C for 2 min 20 s with a final extension at 68°C for 5 min. PCR products were purified by gel extraction (Qiagen Inc, Valencia, CA) and sequenced at the University of Pennsylvania DNA Sequencing Facility.

### DNA immunization and Chikungunya viral challenge studies

BALB/c mice (*n* = 14/each group) were immunized with the pCHIKV-Capsid, the pCHIKV-Envelope (pMCE321) constructs, or control pVax1 (25 µg) 3 times at 2-week intervals, according to a standard DNA immunization protocol. All injections were delivered into the quadriceps muscles in a total volume of 25 µl followed by i.m. electroporation (Inovio Biomedical Corporation, Blue Bell, PA) as described previously [30], [39]. After the last immunization, 4 mice from each group were sacrificed for immunology assays (IFN-γ and Abs ELISA), while the remaining mice (*n = 10*) were used for the challenge studies.

Mice were challenged with 7 log_10_ PFU of the CHIKV isolate (PC-08) by intranasal infection (i.n.) in a total volume of 25 µl and animals were checked daily for clinical signs of infection, such as lethargy and hind limb weakness. Additionally, body weight was monitored [17], [31], [40], [41]. Animals were then sacrificed either 14 days p.i. or earlier if a weight loss of more than 30% was observed.

### Rhesus macaque studies

Non-human primate studies were conducted under a contract at Bioqual Inc, MD. The animal management program of this institution is accredited by the American Association for the Accreditation of Laboratory Animal Care and meets NIH standards as set in the guide for the Care and Use of Laboratory Animals. This institution also accepts as mandatory the PHS policy on Humane Care of Vertebrate Animals used in testing, research and training. Animals were allowed to acclimate for at least 30 days in quarantine prior to any immunization. Four rhesus macaques were immunized at weeks 0, 4, and 8 with 1 mg/construct (at a concentration of 10 mg/ml) of CHIKV envelope (pMCE321) and 3 rhesus macaques were immunized with pVax1 vector. DNA was delivered into the quadriceps muscle (intramuscularly (i.m.) followed by *in vivo* electroporation as previously described [39]. Animals were bled every 2 weeks. Five ml of blood was collected for serum studies and ten ml of blood was collected in EDTA tubes, and peripheral blood mononuclear cells were isolated by standard Ficoll-Hypaque centrifugation and resuspension in complete culture medium (RPMI 1640 with 2 mM/liter L-glutamine, 10% heat-inactivated fetal bovine serum, 100 IU/ml penicillin, 100 µg/ml streptomycin, and 55 µM/liter β-mercaptoethanol). Red blood cells were lysed with ammonium chloride-potassium (ACK) lysis buffer (Invitrogen, CA).

### Neutralization antibody (nAb) and Hemagglutination (HI) assays

The 50% tissue culture infectivity dose (TCID_50_) was calculated and a standard concentration of virus was used for the micro-neutralization test throughout these studies. Microneutralization assays were performed with human patient samples as well as using the mouse sera from pCHIKV-E1/pCHIKV-E2/and pCHIKV-Env (pMCE321) immunized animals, as described previously [42]. Briefly, the patient, mouse, or monkey sera were serially diluted in MEM (1∶10 to 1∶10,240) and incubated with an equal volume of CHIKV (100 TCID_50_) at 37°C. After 90 min, the virus-serum mixture was added to a monolayer of Vero cells (100,000 cells (for patient and mouse samples) and 15,000 cells (for monkey samples) per well) in a 96-well flat bottom plate and incubated for 5 days at 37°C in a 5% CO_2_ incubator. The highest titer at which no CPE was observed was recorded as the nAb titer.

HI assays were performed as described previously for Arboviruses [43] and CHIKV virus isolate was used as the antigen. Kaolin-treated sera from human patient samples or immunized mice were diluted and tested at serial 2-fold dilutions from 1∶10 to 1∶1,280 at pH 6.3, using eight hemagglutination (8HA) units of antigen (CHIKV) and 0.4% goose erythrocytes. The highest dilution of the serum that inhibited hemagglutination was recorded as the HI titer. HI titers greater than or equal to 20 were considered positive [41].

### IFN-γ ELISpot assay and ELISA

ELISpot assays were performed as previously described [33], [39]. Briefly, 96-well ELISpot plates (Millipore) were coated with anti-mouse IFN-γ capture Ab (R&D Systems) and incubated for 24 h at 4°C. The following day, plates were washed with PBS and blocked for 2 h with 1% BSA. Two hundred thousand splenocytes from the pMCE321 Env-immunized mice were added to each well and incubated overnight at 37°C in 5% CO_2_ in the presence of media alone (negative control), media with Con A (positive control), or media with peptide pools (10 µg/ml) consisting of 15-mers overlapping by 9 amino acids and spanning the length of the appropriate protein. After 12 h, the cells were washed and then incubated for an additional 24 h at 4°C with biotinylated anti-mouse IFN-γ Ab (R&D Systems, Minneapolis, MN). Streptavidin–alkaline phosphatase was added to each well after washing and then incubated for 2 h at room temperature. The plates were washed, and then 5-bromo-4-chloro-3′-indolylphosphate *p*-toluidine salt and nitro blue tetrazolium chloride (chromogen color reagent; R&D Systems) were added to the wells. Lastly, the plates were rinsed with distilled water, dried at room temperature, and spot forming units (SFU) were quantified by an automated ELISpot reader (CTL Limited). For each sample, the raw values were normalized to SFU per million splenocytes.

CD8^+^ T-cell depletion studies were carried out following immunomagnetic cell separation. Dynabeads (Dynal Biotech) monkey CD8 (clone BW135/80) was used as the method for separation, resulting in 90% depletion in 20 min using 1×10^7^ beads/ml for 2.5×10^6^ splenocytes. Depletion was conducted as described by the manufacturer. The negatively isolated cells (CD8^+^ T cell depleted) were transferred to a second tube for further use in the ELISpot assays [44], [45].

The proinflammatory cytokines levels following CHIKV infection were determined in the culture medium using a commercially available ELISA kit following the manufacturer's instructions (R&D Systems Inc, MN). All samples were analyzed in triplicate [33].

### Statistical analysis

Data was collected from cellular assays and presented as the mean +/− standard deviation which was calculated from triplicate wells of pooled samples from each experimental group. Prior to all statistical analysis, the normality of the data was confirmed with Levine's test. Analysis between groups was performed using independent samples *t*-test. Comparisons among three groups were performed with ANOVA with a post-hoc Fisher's Least Significant Difference (LSD) test to correct for multiple comparisons between groups. In each case, p≤0.05 was considered to be significant. All statistical analysis was carried out using the Statistical Package for the Social Sciences (SPSS).

## Results

### Clinical observation in CHIKV patients

The clinical manifestations caused by the Chikungunya outbreaks in 2005 to 2007 appeared varied and somewhat divergent from those observed in the early outbreak in 1953. In particular, the hemorrhagic tendency of CHIKV infections is not as predominant as that of past outbreaks [46]. Accordingly, we sought to study serum samples from the recent CHIKV outbreaks and characterize viruses and the humoral responses from acute and convalescent sera of infected patients. During the recent outbreak in India, serum samples were regularly collected from patients with complaints of high fever, chills, headache, vomiting and severe abdominal pain (outpatient department, Sri Ramachandra Medical Centre, Chennai, India & at Regional Medical Research Centre (ICMR), Port Blair, India). From this population, thirty patient samples suspected for CHIKV were randomly selected and included in this study; all of the 30 patients from the outbreak area experienced fever lasting 2–15 days with high-grade temperature (38°C to 40°C), and in some patients (23%) accompanied by chills and rigors. Vomiting and abdominal pain (20% of the patients) as well as headache (13% of cases) was also observed. While myalgia or joint pain was seen only in 3% of the patients, rashes or hemorrhages were not observed in this patient population. Furthermore, sore throat and retro-orbital pain as seen in other common viral infections was not prominent in this outbreak. A summary of the clinical and laboratory observations in this CHIKV-infected patient population is listed in Table 1. Importantly, 13 samples (43%) were RT-PCR positive to CHIKV primers demonstrating that some contained Chikungunya virus.

### Isolation and identification of CHIKV from patient serum

**Figure 1:**
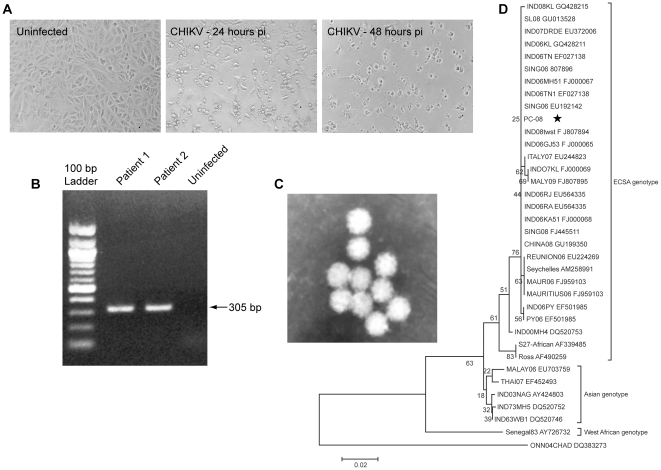
Isolation and identification of CHIKV. The microphotographs show normal uninfected Vero cells (A) and the Vero cells infected with CHIKV virus isolate. CHIKV infection in Vero cells causes characteristic foamy cytopathic effect (CPE) 48 hours p.i. as seen with the isolate. (B) RT-PCR analysis of CHIKV viral isolates. Agarose gel photograph showing the RT-PCR amplified product (305 bp) of the CHIKV positive patient isolates (Lane 1&2). The uninfected negative control (Lane 3) shows no amplification. (C) Electron micrographs of CHIKV viral isolates (D) Phylogenetic Tree generated with E2 amplicon from CHIKV Isolate. * Indicates the PC-08 CHIKV strain.

While samples collected during the first 48–72 h of infection are typically ideal for virus isolation [28], [37], we were able to isolate CHIKV successfully from RT-PCR positive and symptomatic patient samples collected at the 3–4 days post CHIKV infection. Isolation of virus from the serum of a CHIKV positive patient who had a high-grade fever (40°C) lasting for 2 days was verified by the observance of massive cell death (Cytopathic effect: CPE) in Vero cells and by RT-PCR (Fig. 1). As seen in Fig. 1A, the isolated virus induced CPE indicating the presence of infectious CHIKV and successful virus production. To further confirm the identity of the virus that caused CPE, we extracted RNA from the infected cell culture supernatant and performed a one step RT-PCR to amplify a part of the CHIKV E2 gene by reverse transcriptase PCR (Fig. 1B) and electron micrographs of CHIKV viral isolates (Fig.1C) [38]. The E2 gene was selected as the target region for the RT-PCR because this gene shows a high degree of divergence among the alphaviruses [2] and harbors virus-specific nucleotide stretches suitable for primer design. The sequences from the reaction amplicons were then analyzed via a Genbank BLAST search and showed sequence similarity with CHIKV strain Ross (Fig. 1D). This virus was designated as PC-08 (PENN CHIKV strain-2008).

### Construction and expression of CHIKV envelope vaccine

**Figure 2:**
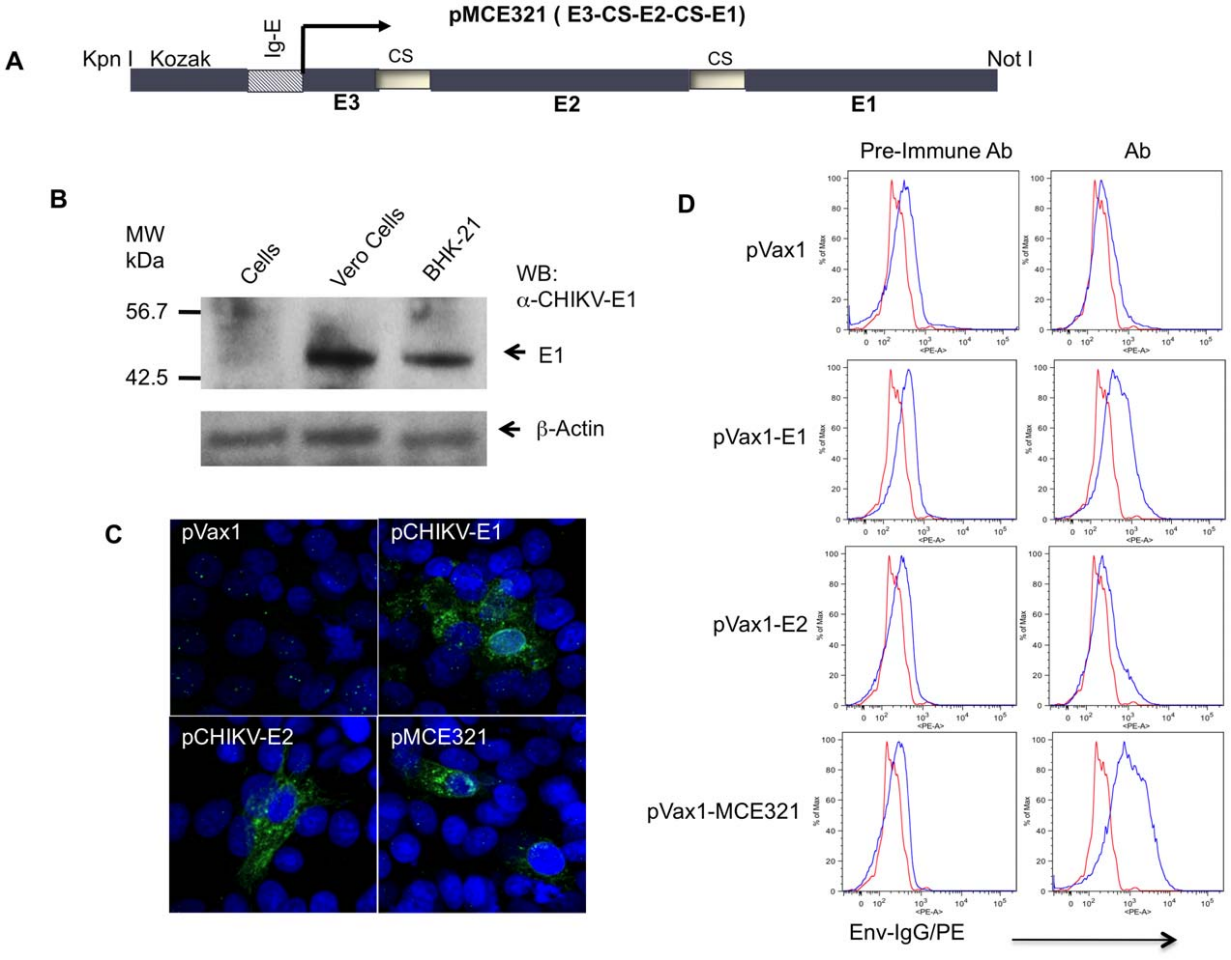
Construction and characterization of CHIKV DNA vaccine. (A) Schematic representation of pMCE321 construct. The flanking enzyme sites used for cloning, Kozak expression element, CMV promoter, human IgE-leader, CHIKV fusion gene (E3-E2-E1), and cleavage sites (CS) are indicated and were cloned into the pVax1 vector. (B) Expression of pMCE321 constructs was confirmed *in vitro* using Envelope-E1 antiserum for the Western blot of CHIKV envelope antigens expressed in Vero and BHK-21 cells by Western blotting. Arrows indicate the positions of E1 protein expression. (C) Immunofluorescent assay showing staining of Vero cells transfected with pCHIKV-E1, pCHIKV-E2, or pMCE321 constructs and transient expression of the envelope proteins. (D). FACS analysis of envelope expression in transfected cells (0.5×10^6^ cells). Vero cells were transfected with indicated constructs and stained with anti-Env sera raised in mice, followed by staining with secondary PE-conjugated anti-mouse IgG antibody as indicated. Two representative FACS histograms are shown.

Previous studies from our laboratory using the envelope E2 and E1 DNA vaccine constructs showed the induction of cellular and humoral responses in vaccinated mice [30]. In the present report, we modified the previous vaccine to optimize its capacity for induction of neutralization Abs by designing a single consensus envelope vaccine construct that expresses all three of the CHIKV envelope glycoproteins (E3+E2+E1). Consensus sequences were optimized for expression, including codon and RNA optimization [30], [47] insertion of a novel leader sequence [36] as well as furin cleavage sites between envelopes to facilitate envelope processing as previously reported [34] and inserted into the pVax1 expression vector and verified by sequencing and designated as pMCE321 (Fig. 2A).

Expression of the pMCE321 vaccine constructs *in vitro* was verified by immunoblotting and immunofluorescence techniques. The vaccine constructs expressed strongly in the transfected BHK-21 and Vero cells and the envelope glycoproteins were detected in the pMCE321 transfected lysates by Western blot using envelope E1 antiserum (Fig. 2B). Further, to evaluate the expression of envelope proteins immunofluorescence techniques were performed with pMCE321 immunized sera in Vero cells transfected with pVax1, pCHIKV-E1, pCHIKV-E2 or pMCE321. Immunofluorescence showed envelope staining of the expressed proteins in the cytoplasm, which strongly suggested immune reactivity to each envelope component of the fusion protein (Fig. 2C). Further, to visualize the expression of pMCE321, pCHIKV-E1 and pCHIKV-E2, we performed a parallel FACS analysis from transfected cells, and studied the surface expression of envelope proteins. Interestingly, the pCHIKV-E1 and E2 expression profile was almost identical (Fig. 2D). Unlike E1 and E2 sera expression, pMCE321 sera respond to strong envelope expression in transfected cells. These findings demonstrated the ability of the pMCE321 construct to potently express in mammalian cells and that the Abs induced by these constructs were able to react with the individual envelope glycoproteins E1 and E2.

### CHIKV envelope vaccine immunogenicity and protection in mice

**Figure 3:**
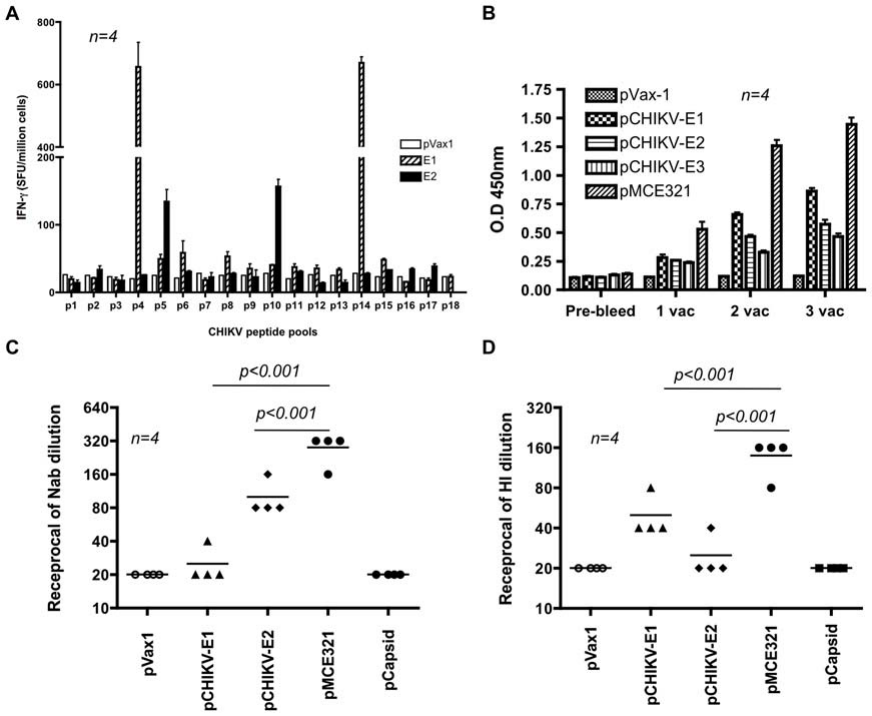
CHIKV DNA vaccination induces strong immunity in mice. BALB/c mice were immunized three times, each 2 weeks apart, with 25 µg pVax1 vector or pMCE321-Env and sacrificed 1 week after the 3^rd^ immunization. (A) Splenocytes from immunized animals were harvested and cultured overnight in the presence of peptide pool matrix spanning the Envelope protein (pool-1 & pool-2) and the IFN-γ response to each pool was measured by ELISpot as described in the Materials and Methods. Values represent the mean and standard deviation of triplicate wells and are representative of three independent experiments. (B) Systemic anti-Env IgG levels after DNA immunization. Each group of inbred BALB/c mice (*n = 4*) was immunized with indicated vaccines. Mice were bled 1 week after each immunization, and then sera were diluted to 1/100 for reaction with CHIKV-Env. OD was measured at 450 nm. Values and bars represent mean (*n = 4*) and the SEM. (C and D) Quantification of CHIKV specific neutralizing and HI titer in sera from DNA immunized mice (pVax1/pCHIKV-E1/pCHIKV-E2 and pMCE321) to CHIKV. The nAb titers are plotted as the highest dilution of serum that resulted in at least 50% inhibition of CPE. The highest dilution of the serum that inhibited hemagglutination was recorded as the HI titer. Similar results were observed in three independent experiments with at least *n* = 4 per group for each experiment.

The ability of pMCE321, to induceCD8^+^ CTL responses in mice after three immunizations was determined by IFN-γ ELISpot assay. Expression of all three of the envelope glycoproteins from the single DNA vaccine construct induced detectable cellular immune responses against CHIKV envelope specific peptide pools (Fig. 3A). The results of the IFN-γ ELISpot assay 1 week following the third i.m. immunization (mean count, 1,613±117) for pMCE321 against both peptide pools showed strong cellular immune responses to administered envelope Ag in contrast to the control. pCapsid- or pVax1-immunized mice showed no envelope-specific Ag-specific responses at any point during the study. Capsid-specific T cell responses were induced to the Capsid vaccine. Because of increased T cell responses to the combined CHIKV-Envelope vaccine observed in mice, we anticipated that the Ab responses to combined envelopes would be similarly increased. To examine this, sera collected one week after each immunization were tested by ELISA to detect the induction of Envelope-specific IgG. Interestingly, mice immunized with the pCME321 complex displayed significantly higher levels of envelope-specific serum IgG than mice immunized with E1, E2 or E3 alone (Fig. 3B). As expected, the control plasmid, pVax1 did not elicit any detectable Ab responses as determined by ELISA.

The combination of CHIKV envelope glycoprotein genes into one vaccine construct, pMCE321, induced measurable levels of neutralizing and HI antibodies which are significantly greater than responses induced by pCHIKV E1 and E2 constructs alone (Fig. 3C–D; *p*<0.001). Interestingly, we also observed that the E2 and E1 constructs when delivered individually were able to induce high levels of neutralizing Ab responses or HI titers respectively, but not both showing segregation of these functions. Importantly, the pMCE321 construct was able to drive both neutralizing and HI Ab responses at levels higher than either construct on its own. Taken together, these data demonstrate that immunization with the pMCE321 DNA vaccine induced both cellular and humoral immunity in mice.

**Figure 4:**
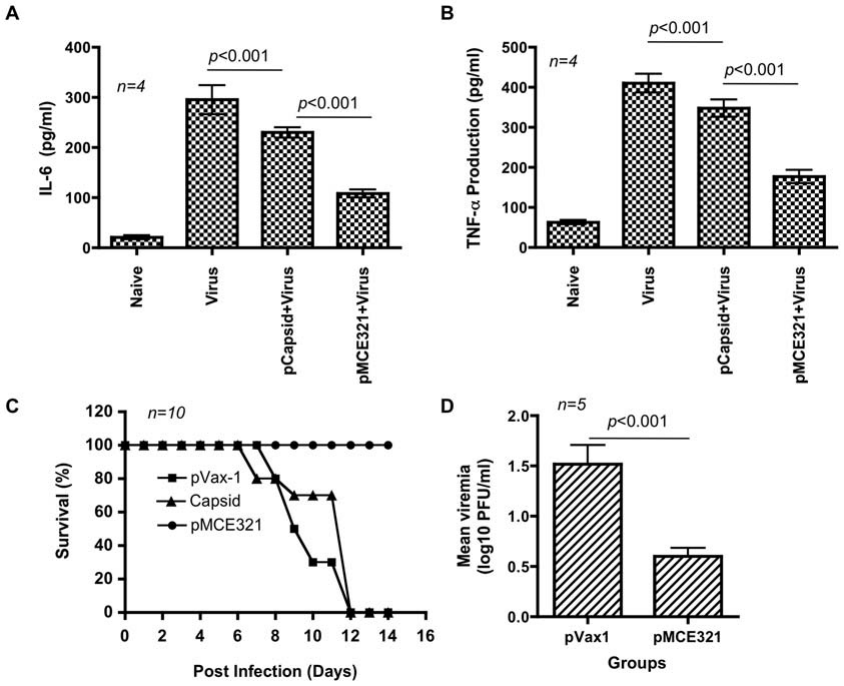
CHIKV DNA vaccination and infection. (A&B) Analysis of proinflammatory cytokines (TNF-α and IL-6) in CHIKV vaccinated and infected mice. Cytokine levels (pg/ml) were assayed by ELISA from the cell free sera from 10 days post infection. These data represent the average 3 wells/mouse and standard deviations of 4 mice. (C) Percent survival in CHIKV-challenged mice. Similar results were observed in 2 independent experiments with at least *n* = 10 per group for each experiment. (D) The viremia, 5 day after challenge, as measured by a plaque assay. Mice immunized with pVax1 (control) or immunized pMCE321 (vaccine) were challenged with the PC-08 CHIKV strain at a dose of 7 log10 PFU by the intranasal route. Data are mean ± SEM of 5 animals.

We next addressed whether levels of CHIKV vaccine-elicited immunity were able to confer protective immunity by virtue of its cellular and neutralizing/HI Ab responses in mice. A virus challenge study was conducted to assess protective efficacy of the pMCE321 envelope vaccine in comparison with a CHIKV-Capsid vaccine which induced cellular responses, but no neutralization or HI responses. CHIKV-challenged mice were monitored daily for 14 days p.i. and outcome of the challenge was evaluated based on the common signs of Chikungunya infection in mice such as reduction in body weight, survival, lethargy, and hind limb weakness reported in previous studies [31], [41]. A recent study by Ng *et al*., strongly suggested that proinflamatory cytokines such as IL-1β, TNF-α and IL-6 are biomarkers that have utility in measuring disease severity during CHIKV viral infection [48]. Therefore we also analyzed the production of these pro-inflammatory cytokines in naïve and vaccinated mice post infection and compared the levels to naïve-uninfected mice. Neither IL-6 nor TNF-α were detected in significant amounts in naïve mice; conversely, the production of IL-6 and TNF-α in CHIKV-infected mice (virus) was significantly greater than that in naïve mice (Fig. 4A–B). Similarly, secretion of proinflammatory mediators including IL-6 and TNF-α was measured in vaccinated mice and found to be strongly activated in pCapsid-immunized mice. In contrast remarkably lower levels were detected in pMCE321-immunized mice (Fig. 4A–B) (*p*<0.001 versus pCapsid). These data suggest that CHIKV Envelope DNA immunization, and not Capsid vaccination, was more effective in minimizing the secretion of proinflammatory cytokines that are commonly associated with viral pathology.

Further, signs of disease in the naïve and vaccinated mice infected with the CHIKV isolate were detected and all mice showed a reduction in body weight for a period of 3 days p.i. However, after this initial period, pMCE321-immunized animals exhibited a restoration in body weight on average when compared with either naïve or Capsid-vaccinated animals. While none of the animals died naturally due to infection, the Capsid-immunized and naïve groups continued to lose body weight and were subsequently euthanized over the period of 7–12 days p.i when their body weight loss was greater than 30% of the pre-challenge weight (Fig. 4C). In contrast 100% of the pMCE321 vaccinated animals recovered and survived beyond day 12 p.i. Viremia on day 5 after i.n. infection was also measured in five unvaccinated animals infected with the CHIKV virus (mean titer log_10_ 1.5 PFU/ml ±0.42), and vaccinated animals (mean titer log_10_ 0.58 PFU/ml ±0.17). The vaccinated animals had significantly lower viremia (*p*< 0.001) with no signs of infection and remained apparently healthy (Fig. 4D).

### Histopathologic evaluation of CHIKV-challenged mice

Histopathological studies were conducted in vaccinated and naïve animals in the brain for neurological manifestations, liver (the initial site of virus replication), lungs (the portal of entry alternate to skin), heart and kidneys.

#### Brain

**Figure 5:**
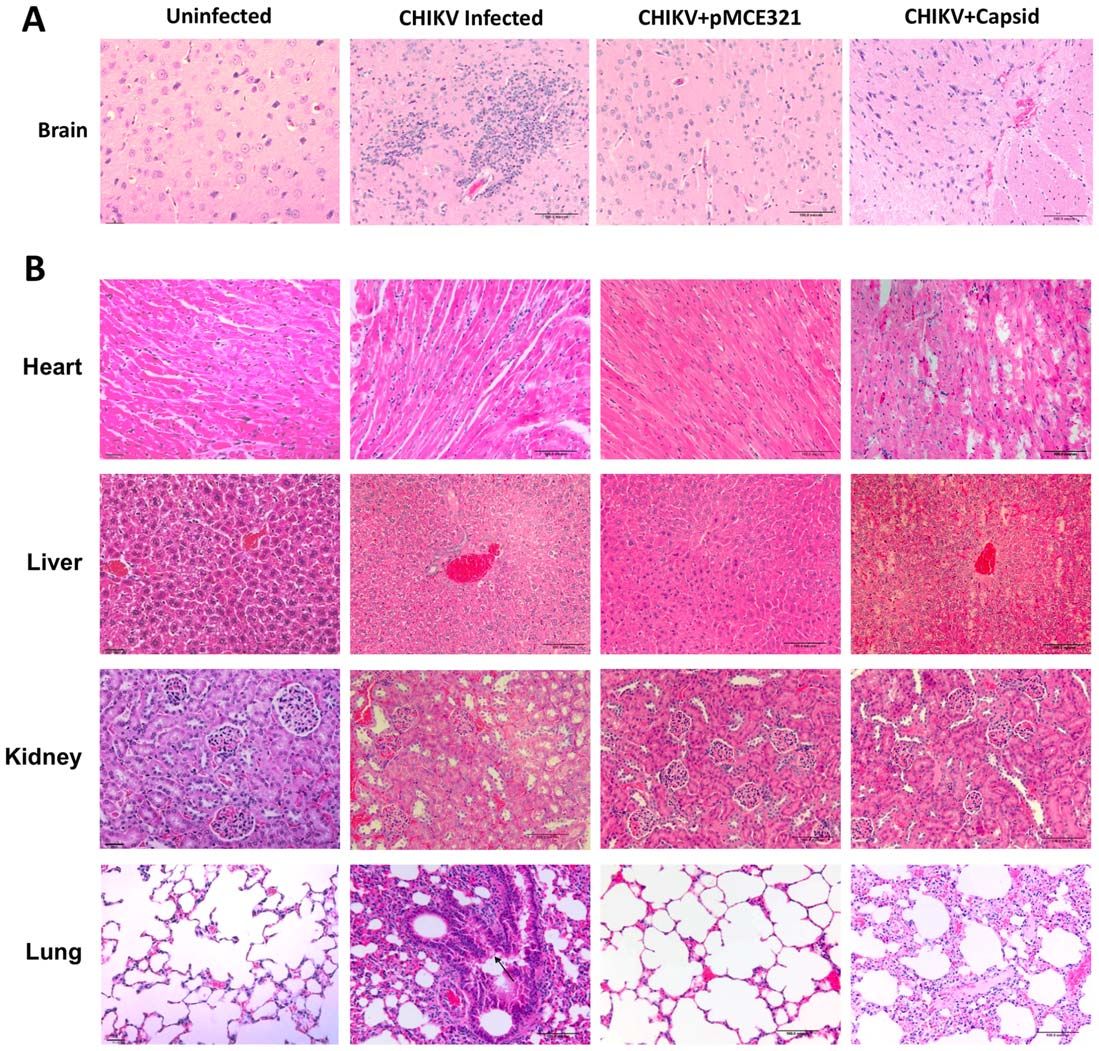
Histopathology analysis of CHIKV challenged mice. (A) H&E stained sections of Brain. pMCE321 vaccinated mice showed no severe pathological changes and showed only minimal microglial formation. Capsid immunized mice group showed severe hemorrhage and microglia formation similar to the naïve group. (B) H&E stained sections of Heart, Liver, Kidney and Lungs. Envelope vaccinated group showed minimal or no pathological changes in the organs. The naïve group showed severe pathological changes indicative of virus infection and the Capsid DNA immunized group showed similar pathological changes to the naïve group. Representative data are shown from 2 mice/group.

The CHIKV-infected brains of the naïve mice showed severe spongiform changes and a large number of apoptotic bodies and microglial nodule formation in the external granular layer of the cerebral cortex. We also observed moderate edema. The Capsid-vaccinated mice showed severe hemorrhage in cerebral cortex and microglial infiltration in lamina pyramidalis externa. Modicum microglial and minimal edema was seen in the CHIKV-infected brains of pMCE321-immunized mice suggesting immunization decreased CHIKV-mediated pathology of the brain (Fig. 5A).

#### Heart

The histopathological analysis of the heart tissue in naïve mice showed severe myocardial degeneration/necrosis. Also, infiltration of inflammatory cells between myocardial fibers was seen. Most of the myocardium was destroyed in Capsid-immunized mice with punctuated hemorrhage. A compact and orderly arrangement of myocardium was evident in the pMCE321-immunized mice and nuclei of the cells were clearly visible (Fig. 5C).

#### Liver

As expected in the CHIKV-infected naïve group, severe degeneration/necrosis presented in the centrilobular region. Dilation and hyperemia of sinus hepaticus, lymphocytic infiltration, and thrombus formation in the lumen was also seen in this group. Hepatocyte vacuolar degeneration and focal necrosis around the central vein were observed in the Capsid-immunized group. Hepatic sinus dilation and lymphocytic infiltration were seen in the Capsid-immunized group. In contrast the pMCE321-immunized group did not show any severe pathologic abnormalities (Fig. 5C).

#### Lungs

In the CHIKV-infected naïve mice, bronchiole epithelial cell exfoliation, and inflammatory infiltration around bronchioles were observed. Furthermore, alveolar sacs were observed to be filled with exfoliative and inflammatory cells. A similar pathology was observed in the capsid-immunized group, in addition, and an alveolus filled with red blood cells and thrombosis was observed in the pulmonary venule. In contrast, no significant pathologic changses were observed in the lungs of CHIKV-infected pMCE321-immunized mice (Fig. 5C).

#### Kidney

Microscopic observation of sections using hematoxylin-eosin staining revealed degeneration/necrosis of renal tubular cells in the naïve mice, which caused renal tubular lesions. Hyaline casts were observed. Also, severe hemorrhage and inflammatory cell infiltration was seen between the renal tubules. In the Capsid-immunized mice, renal tubular cells showed severe degeneration/necrosis, more hemorrhage and infiltration of inflammatory cell. However, in the pMCE321-immunized mice very few renal tubular cells degenerated, hemorrhage and inflammatory cells infiltration was minimal compared with those of the naive group (Fig. 5C).

### CHIKV DNA vaccination is immunogenic in nonhuman primates

**Figure 6:**
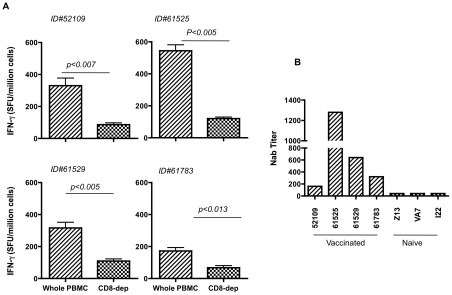
Immunogenicity of CHIKV DNA vaccine in nonhuman primates. (A) IFN-γ ELISpot assay results presented are from individual macaques 2 weeks after the fifth immunization against pMCE321 administered vaccine. PBMCs harvested from animals immunized with pMCE321 were used in the IFN-γ assay. We also tested PBMCs that were depleted of CD8^+^ T cells by magnetic bead separation before *in vitro* stimulation. The PBMCs were incubated in the presence of the following stimulators and controls: R10 medium (negative control), Con A (5 µg/ml positive control), and 10 µg/ml CHIKV peptide mix. Data are presented as the SI (experimental counts/spontaneous counts), where the spontaneous count wells are from the R10-negative control wells as described in Materials and Methods. Values represent the mean of triplicate cultures and are representative of three independent experiments. (B) nAb titers from sera of DNA vaccinated monkeys are shown. The pMCE321 DNA vaccine construct induced nAb responses ranging from 80-1,280 titers and mimicked those induced in convalescent patient sera.

Nonhuman primate studies were performed to determine whether CHIKV DNA vaccination with pMCE321 could elicit cellular as well as humoral responses, characterized by the elicitation of nAb responses. Four rhesus macaques were vaccinated with pMCE321 DNA delivered by *in vivo* EP. As negative controls, three monkeys were vaccinated with the pVax1 control vector. The animals were then monitored for the development of CHIKV Envelope-specific CD8^+^ T-lymphocyte and nAb responses. Two weeks after the fifth DNA immunization, cells and serum were collected and tested for immunogenicity. Three of the four CHIKV pMCE321 DNA-immunized monkeys had detectable envelope -specific functional CTL activity as measured by IFN-γ ELIspot (Fig. 6A). The control plasmid-immunized monkeys remained negative throughout the course of the experiment. Furthermore, of the four monkeys immunized with the pMCE321 DNA vaccine, all four monkeys developed nAb titers. These averaged 570 and ranged from 80-1,280 titers (Fig. 6B).

### CHIKV induces neutralizing antibodies in humans and correlates with HI-Ab response during infection

**Figure 7:**
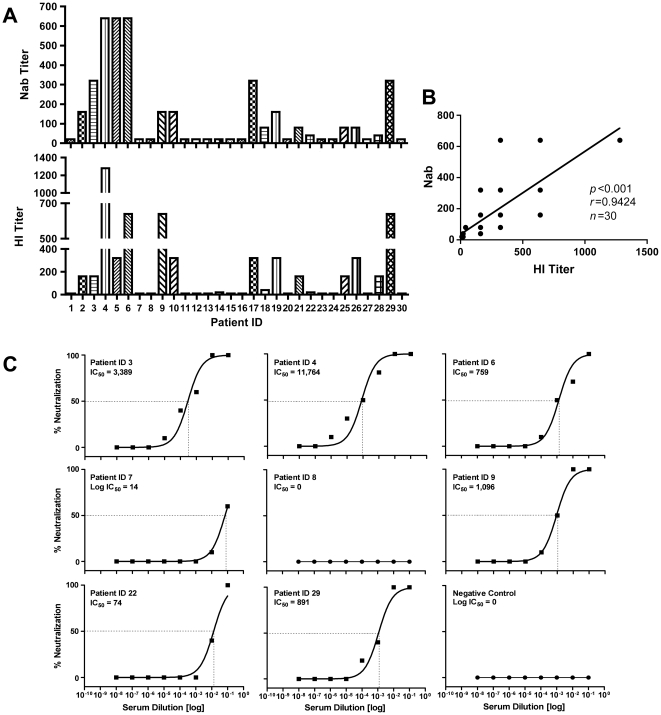
Antibody-mediated neutralization and Hemagglutination Inhibition from CHIKV-infected patient serum. nAb titers (A) and Hemagglutination Inhibition (HI) antibody responses (B) in patient sera (SRMC-1 to SRMC-30) to CHIKV. Similar results were observed in 2 independent experiments. There is a positive correlation exists between nAb and HI on CHIKV infected patients (C). These relationships were evaluated using the Spearman correlation test using the Prism 5 Graph Pad software. Neutralization of CHIKV infectivity with patient serum (D). The IC50 is defined as the reciprocal of the antiserum dilution at which CHIKV virus entry is 50% inhibited (dashed line). Similar results were observed in 2 independent experiments.

We next compared macaque nAb titers with those observed in human patient convalescent sera. Understanding the correlates of immunity during CHIKV infection is likely important for the rational design and development of an effective vaccine. While it is clear that a nAb response appears to be critical for protection against CHIKV infection, as with many infectious diseases like influenza and hepatitis, the levels required to induce sterilizing immunity or protection from disease-related morbidity are currently unknown. Therefore, we tested serum samples from CHIKV-infected individuals to measure levels of nAb activity. Among the thirty patients tested in this study, sixteen patients (53%) showed nAb titers to CHIKV, (titers above 20 titers were considered as positive; [41]), ranging from 40–640 titers (Fig. 7A). Further, the presence of HI Abs to arboviruses including CHIKV was observed in seroepidemiological studies [18], [32]. However the importance of HI during active CHIKV infection is not well understood. During the recent outbreak investigation, HI Abs were observed in eighteen (60%) of the thirty patients tested in the study. The HI titers varied from 20-1,280 titers (Fig 7B). Indeed, the HI titer also directly correlated with the levels of nAb in CHIKV infected patients (*r* = 0.9424; *p*<0.001) (Fig. 7C), suggesting that the neutralizing and HI antibodies to CHIKV correlate with the ability of the host to clear the infectious virus during the course of natural infection. The neutralization titer was defined as the highest dilution of serum that prevented virus propagation as determined by CPE (Fig. 7D). Interestingly, the post-infection nAb titers in convalescent humans were in the same range as the vaccine- induced titers we observed in macaques suggesting its value as a potential model for CHIKV vaccine development.

The mouse and primate immunological data taken together demonstrate that vaccination with pMCE321 induces a strong CD8^+^ T cell-mediated cellular response as well as a humoral response (nAb and HI titers) capable of protecting the animals against a lethal challenge. In convalescent human samples we report the presence of significant nAb titers. Taken together, the data is suggestive of the role of antibody responses in protecting against CHIKV.

## Discussion

CHIKV is an emerging pathogen and an important public health concern [2], [5], [12], [13], [49]. Since no licensed vaccine or treatment is available for the pathogen, there is an urgent need for an effective vaccine [30]. In this report, we describe the development of a DNA vaccine construct from our laboratory that expresses three of the CHIKV envelope proteins (E3, E2, and E1), is immunogenic in mice and nonhuman primates, provides protection in mice, and drives neutralizing titers in primates similar to those observed in human patient convalescent sera.

During the recent outbreak in India, sera were obtained from human patients with suspected CHIKV disease. All patients were from outbreak areas and a total of thirty sera were randomly selected and included in this study. However, the clinical picture reported herein had a different pattern with respect to previous reports [50]; this cohort had less severe disease symptoms and the reasons attributed to this could be multifactorial, such as the time of sample collection, the magnitute and types of immune responses mounted by these individuals, and the presence of pre-existing immunity in the outbreak area. RT-PCR confirmed the laboratory diagnosis and most of the patients in the study group were found to be positive, additionally having neutralizing/HI Abs to CHIKV. Virus isolation was successfully accomplished from the serum of a febrile patient and the virus isolate was identified and confirmed as CHIKV by RT-PCR amplifying the partial region of the E2 gene. Further, phylogenetic tree analysis with this sequence revealed a similarity to the Chikungunya strain Ross.

The importance of nAb against a viral infection has been well established and was reported earlier in a recent CHIKV study and also an RRV study, a virus that is similar to CHIKV [32], [51]. Viral clearance was associated with the rapid induction of nAb in the acute phase of infection and loss of nAb after recovery from infection [52]. The same concepts may also extend to immunity to CHIKV infection. For example, previous reports in populations with high levels of nAb against CHIKV showed low infection rates, possibly due to protective immunity due to prior exposures, thus resulting in subsequent protective immunity [2], [17], [48]. For instance, a study in northern Malaysia found that 35% of adults had nAbs against CHIKV even though there were no reports of a CHIKV outbreak in Malaysia, during this period of time. Furthermore, serologic surveys in India not linked to a recognized outbreak found a prevalence of 4.4% in the Calcutta metropolis and 15.3% in Andaman and Nicobar Island [7], [16], [53]. Hence, it is likely that nAb aid in the reduction of symptoms either by aiding in clearing the virus or by preventing the pathological damage caused by the virus. In the current study, the range of nAb titers observed in the study cohort ranged between 40 and 640. Similarly, the HI titers induced in patients were in parallel with the neutralizing titer with HI titers ranging from 20 to 1,260.

The findings of significant nAb titers in human patient sera during active infection encouraged us to compare the capacity of our CHIKV DNA vaccine to generate nAb. Our previous CHIKV DNA vaccine consisting of the co-delivery of two different plasmids, pCHIKV-E1 and pCHIKV-E2, was capable of inducing levels of neutralizing and HI titers; pCHIKV-E2 induced the production of nAb and pCHIKV-E1 elicited HI Abs response in immunized mice. These findings led us to combine the predominant genes that constitute the entire CHIKV envelope in a single envelope construct (pMCE321) for vaccination. We envisioned such an approach may lead to simultaneously increased cellular responses and humoral responses and likely inducing both neutralizing and hemagglutination inhibition Abs. Indeed, the novel envelope construct pMCE321 vaccine drove the cellular response to both E1 and E2 glycoproteins and the magnitude of this response was higher than that seen with individual E1/E2 gene constructs. Moreover, the humoral responses to pMCE321 were also increased when compared to that of the individual pCHIKV-E1 and pCHIKV-E2 constructs.

To assess the protective efficacy of this novel vaccine construct capable of generating both strong cellular and humoral immunity, we conducted a CHIKV challenge study in mice and observed that the PC-08 strain of virus was pathogenic in mice. Specifically, we observed a reduction in body weight, lethargy, hind limb weakness and high levels of proinflammatory cytokines such as IL-1β, TNF-α and IL-6 [31], [40]. Following challenge with CHIKV, all of the naïve mice showed severe weight loss from 1-3 days p.i. and showed clinical symptoms like lethargy and hind limb weakness by day 6. However, none of the envelope-based vaccine-immunized mice showed clinical symptoms as pronounced as in the unvaccinated mice. While control mice continued to lose weight over the course of the study and had to be euthanized, the Env-immunized mice rapidly regained weight after the initial 3 days and returned to their normal, pre-challenge state. Overall, these data showed that the new envelope-based vaccine construct pMCE321 was highly effective at protecting against morbidity and mortality in this model. Furthermore, our study also demonstrated an inverse relationship between vaccination and the resulting viremia. Interestingly, we observed a reduction in the amount of virus in the vaccinated mice compared to the unvaccinated mice post-challenge. Similarly, the histopathological evaluation of tissues from the brain, liver, heart, lung and kidney in immunized mice showed minimal or no damage when compared to naïve infected mice. Furthermore, Capsid-immunized mice exhibited symptoms of morbidity as well as mortality likely due to the lack of induction of nAb responses. This protective efficacy may likely be attributed to the high titers of nAb produced in the vaccinated animals similar to the findings in humans where high neutralizing titers have been correlated to better disease prognosis and protection [15], [17], [32], [54].

Due to the induction of strong immunity and protection in mice, we next wanted to assess whether the envelope DNA vaccine (pMCE321) was immunogenic in nonhuman primates. Four rhesus animals were immunized and the cellular and humoral responses were measured. Similar to the immune responses observed in vaccinated mice, CHIKV envelope-specific T cell responses were induced as well as nAb responses in the nonhuman primate cohort. Importantly, the range of neutralization titers observed in these animals was similar to the levels observed in humans during active CHIKV infection. These data demonstrate that the pMCE321 DNA vaccine is immunogenic in nonhuman primates, and was capable of producing titers of nAb which are thought to be protective in humans against disease.

In summary, there are several important findings in this manuscript. We report the isolation of a new isolate of CHIKV from the southern regions of India which we have named PC-08. This isolate is cytopathic in several cell lines as well as primary immune cells. The virus can induce pathogenesis in a mouse challenge model through i.n. challenge and thus should provide a useful *in vivo* model for further study. Furthermore, this viral stock allowed us to scale up a Neutralization assay for CHIKV study. We also report development of a novel single-plasmid envelope-based DNA vaccine; pMCE321 is protective in the mouse challenge model and drives relevant titers of nAb in a macaque model system. Further study of this novel vaccine and protective immunity is warranted.
